# Studies of Cellulose and Starch Utilization and the Regulatory Mechanisms of Related Enzymes in Fungi

**DOI:** 10.3390/polym12030530

**Published:** 2020-03-02

**Authors:** Bao-Teng Wang, Shuang Hu, Xing-Ye Yu, Long Jin, Yun-Jia Zhu, Feng-Jie Jin

**Affiliations:** College of Biology and the Environment, Co-Innovation Center for Sustainable Forestry in Southern China, Nanjing Forestry University, 159 Longpan Road, Nanjing 210037, China; wangbaoteng123@163.com (B.-T.W.); Hushuang163njfu@163.com (S.H.); yuxy1995@163.com (X.-Y.Y.); isacckim@kaist.ac.kr (L.J.); zhuyj@njfu.edu.cn (Y.-J.Z.)

**Keywords:** fungi, polysaccharides, enzyme, regulator, cellulase, amylase

## Abstract

Polysaccharides are biopolymers made up of a large number of monosaccharides joined together by glycosidic bonds. Polysaccharides are widely distributed in nature: Some, such as peptidoglycan and cellulose, are the components that make up the cell walls of bacteria and plants, and some, such as starch and glycogen, are used as carbohydrate storage in plants and animals. Fungi exist in a variety of natural environments and can exploit a wide range of carbon sources. They play a crucial role in the global carbon cycle because of their ability to break down plant biomass, which is composed primarily of cell wall polysaccharides, including cellulose, hemicellulose, and pectin. Fungi produce a variety of enzymes that in combination degrade cell wall polysaccharides into different monosaccharides. Starch, the main component of grain, is also a polysaccharide that can be broken down into monosaccharides by fungi. These monosaccharides can be used for energy or as precursors for the biosynthesis of biomolecules through a series of enzymatic reactions. Industrial fermentation by microbes has been widely used to produce traditional foods, beverages, and biofuels from starch and to a lesser extent plant biomass. This review focuses on the degradation and utilization of plant homopolysaccharides, cellulose and starch; summarizes the activities of the enzymes involved and the regulation of the induction of the enzymes in well-studied filamentous fungi.

## 1. Introduction

Polysaccharides are relatively complex carbohydrates that are widely distributed in nature. They are biopolymers made up of a variety of monosaccharides joined together by glycosidic bonds. Plant polysaccharides are the most abundant carbon source and can be divided into plant cell wall polysaccharides (such as cellulose, hemicellulose, and pectin) and storage polysaccharides (such as starch and inulin) [1,2,3].

All plant cells are surrounded by complex cell walls, and secondary cell walls form the architecture of plant biomass. Different plant cell wall polysaccharides are interconnected with each other and linked to the aromatic polymer lignin to provide the mechanical strength and structural integrity of plant cells. Among them, cellulose fibrils are synthesized at the plasma membrane, while hemicelluloses and other matrix polysaccharides are produced in the Golgi apparatus [4]. The final step of secondary cell wall formation is lignification, which is caused by monolignol secretion by the lignifying cell and/or neighboring cells [5,6]. Lignin polymer deposition in the apoplast provides physical and chemical recalcitrance to plant tissues through the formation of lignocellulosic complexes [4]. In addition to polysaccharides and lignins, plant cell walls also contain several types of structural proteins, such as arabinogalactan proteins, extensins, and lectins [7,8].

Cellulose, as the major component of plant biomass, is the most abundant polysaccharide in the world. Cellulose is a linear polymer consisting of β-1,4-linked D-glucose residues. These glucose chains are tightly bonded by hydrogen bonds to form insoluble fibrous materials. The cellulosic polymer has been described by a two-phase model, consisting of crystalline and amorphous phases often interrupted by a series of semicrystalline structures, which makes it difficult to be utilized by active carbohydrate enzymes [9]. Compared with cellulose, another major component of plant cell wall polysaccharides, hemicelluloses, are more diverse and complex heterosaccharides, which are derived from a heterogeneous group of sugars including D-xylose, D-galactose, and D-mannose. Among them, the most abundant hemicellulose is xylan, whose backbone is a chain of β-1,4-linked D-xylose residues [10]. Relative to cellulose, hemicellulose has a smaller molecular weight and can be dissolved in alkaline solutions.

As one of the most abundant natural resources, cellulose has been used in many different ways, such as in industrial fermentation, fiber material, and papermaking. As a polysaccharide, cellulose has been regarded as an important cornerstone of developments in bioenergy [11,12,13,14,15]. At present, using lignocellulose biomass as a carbon source substrate to produce methane, ethanol, and biofuels by microbial fermentation has become a hotspot in renewable energy research [16,17,18]. In addition, the cellulose utilization of microorganisms also has an important effect on the carbon cycling process, which is one of the largest material flows in the biosphere. Crop straw is a valuable bioenergy resource in agroecosystems. To date, the crop straw industry treatment is still in a compromised state, with high energy consumption, high pollution, low output, and low efficiency; therefore, the comprehensive utilization of crop straw resources will be of great significance to promote resource conservation, environmental protection, and sustainable agricultural development. Recently, some studies have shown that crop straw can be used as a substrate for fermentation to produce biogas or methane [19,20]. Bioenergy research on the production of biogas from lignocellulose biomass through anaerobic fermentation has great potential but has not been widely adopted. Because of its complex structure, lignocellulose is not easily decomposed and utilized by microorganisms [21]. The slow degradation rate of lignocellulose seriously affects the reaction time of anaerobic digestion in biogas production and undermines the economic feasibility. Therefore, in the past few decades, various physical, chemical, and biological pretreatment technologies have been developed for the better use of lignocellulose biomass to obtain high-yield biogas [22,23,24]. Most of the pretreatments for plant biomass are directed to get rid of lignin, which is the main polymer that hampers cellulose and hemicellulose utilization, and many of them simultaneously disrupt the other polysaccharides in the cell wall. However, these studies were mostly based on the pretreatment of the whole corn stalk [21,25], and it is difficult to unify the pretreatment conditions because the chemical components of different parts of the corn stalk are quite different. In addition, physical and chemical pretreatment methods can also cause serious energy consumption and environmental pollution [26]. Therefore, it is an important task for us to further improve the fermentation capacity of microorganisms by increasing the activities of enzymes related to lignocellulose biomass decomposition and optimizing the cultivation conditions of microorganisms. In addition, the gradually increasing global energy crisis requires us to further develop and explore new bioenergy and other renewable energy sources, such as the use of lignocellulose biomass resources by microbial anaerobic fermentation [27]. Another application of cellulose is nanocellulose materials, which are nontoxic, biodegradable, and biocompatible and have no adverse effect on the environment and human health. Because of their good physical and chemical properties, nanocelluloses are widely used in thermoreversible hydrogels, food packaging, flexible screens, coating additives, paper, optical transparent films, and biopharmaceuticals, for example [28,29,30,31].

In recent years, as increasing numbers of cars are produced and used in the world, the demand for vehicle fuel is expanding annually. Some studies have shown that ethanol can be used as an alternative fuel [32,33,34]. The production of ethanol from plants has been known since ancient times, but its substrate is amyloid polysaccharides. The major amyloid polysaccharide is starch, which consists of multiple glucose units that are linked by α-1,4-glycosidic bonds and branched by α-1,6-glycosidic bonds. As an important plant storage polysaccharide, the main sources of starch are cereal grains, which are widely used in traditional food fermentation production, such as liquor and soy sauce, for example [35,36]. Recent studies have shown that starch granules can also be used to prepare nanoscale starch particles, which have unique physical properties. Because starch is an environmentally friendly material, starch nanoparticles are considered to be a promising new biomaterial for use in foods, medicines, cosmetics, and various composite materials [37].

In this review, we discuss recent advances in the utilization of major plant polysaccharides (cellulose and starch), related enzyme production, and their molecular regulatory mechanisms in fungi. We aimed to better understand the degradation of plant polysaccharides and the regulatory mechanisms of related enzymes that can help us to acquire better strains that are more suitable for industrial fermentation utilization.

## 2. The Fungi and Their Potential in the Utilization of Plant Polysaccharides

The filamentous fungi of the genus *Aspergillus* could release large amounts of conidia and are widely distributed in the environment, such as soil, grain, and organisms. *Aspergillus* species, as important environmental microorganisms, play important roles not only in the traditional food fermentation industries but also in the growth and development of plants and animals because of their strong capacity for the decomposition of organic matter and pathogenicity. Some *Aspergillus* species, such as *Aspergillus niger*, *Aspergillus awamori*, and *Aspergillus oryzae*, are used to produce a variety of fermented products, which are mostly related to liquor, vinegar, and soy sauce [35,38,39]. Among them, *A. oryzae* is an industrially important filamentous fungus, which could utilize the cereal grain to produce traditional fermented foods such as liquor [40,41]. *A. oryzae* has the ability to produce and secrete large amounts of amylase, which could break down starch into glucose. Glucose can be further degraded into low-carbon sugar through the glycolysis pathway, and finally converted into ethanol by anaerobic fermentation. At the same time, *A. oryzae* can also secrete large amounts of proteolytic enzymes essential for soy sauce production [42]. Another filamentous fungus, *A. niger*, is widely used in the industrial production of citric acid and gluconic acid because of its ability to secrete organic acids in large quantities [39,43]. In recent years, using starch as an inducer and a strong starch-inducible promoter, some *Aspergillus* species have also been used widely in the efficient production of heterologous proteins, including various active enzymes, and even some heterologous proteins derived from higher eukaryotes, such as plants and animals [44,45,46].

In addition to the *Aspergillus* species, some yeast strains are also often used for alcohol fermentation in industrial production. For example, the yeast *Saccharomyces cerevisiae* can convert polysaccharides into ethanol and some other vital metabolites, such as acetate, glycerol, pyruvate, succinate, and esters. Some of the most significant food and beverages known to humans (such as beer, wine, and bread) are made through the alcoholic fermentation process. These traditional fermentation products are produced by using yeast strains to spontaneously ferment carbon-rich substrates (e.g., bread/beer from cereals, wine from grapes) [36,47,48,49]. Some studies have shown that despite many kinds of microbes involved in the brewing of wine and other fermentation production, *S. cerevisiae* has always been the dominant species [50,51,52]. The yeast population dynamics of winemaking follow a consistent growth pattern, in which a large number of non-*Saccharomyces* initially appear in the early stages but are soon replaced by *S. cerevisiae* strains to finish the fermentation production. The advantage of *S. cerevisiae* over other yeast species in alcoholic fermentation has traditionally been attributed to its high fermentation capacity and adaptation to harsh environmental conditions [53]. Although these fungi can use starch from grains to produce alcohol, this substrate is too expensive to be used to produce alcohol as an alternative to biofuels and can cause serious food waste. Therefore, it is urgent to use lignocellulosic biomass as a carbon source to produce alcohol and other bioenergy efficiently.

Plant biomass is the most abundant renewable carbon resource on earth, and the bioconversion of plant polysaccharides has already attracted extensive attention due to the potential applications as described above. Plant biomass is degraded and utilized by a variety of microorganisms that play crucial roles in the carbon cycle of the ecosystem [54]. Among them, the filamentous fungi *Trichoderma reesei* is an effective cellulase-producing strain and the most studied cellulose-decomposing fungus. The filamentous fungus *T. reesei* is an ascomycete that can grow rapidly and is widely distributed in soil environments. It was originally isolated from the South Pacific [55] and is well known for the ability to secrete large amounts of cellulase, especially when cellulose is used as the carbon source. To date, a large number of studies have deeply explored not only the function of glycoside hydrolase but also the molecular mechanism of regulation of related enzyme–gene expression in *T. reesei* [56,57,58,59,60]. Due to its industrial importance and the multiple uses of cellulase in *T. reesei*, many mutants that increase cellulase yield have been acquired through conventional mutagenesis techniques. Currently, some mutants have been reported to secrete high yields of cellulase into the medium for industrial utilization [61,62,63]. In addition to *T. reesei*, there are also some other microorganisms that can use cellulose as a carbon source to produce useful substances. For examples, several wood-rotting basidiomycetes, white rot and brown rot fungi, some plant pathogens, the basidiomycetous yeast *Rhodotorula glutinis*, etc. have been isolated [64,65,66]. Basidiomycetes are the most potential cellulose degraders since many species grow on dead wood or litter, in environments rich in cellulose, and they have been studied extensively [67]. Different strains are suitable for different fermentation industries, and researchers are constantly trying to choose better ones to exploit [68,69]. Recent studies also showed that the saccharification of wheat straw was importantly enhanced by mixing enzymes from *T. reesei* and *Aspergillus* species [70,71]. Cocultivation of multiple fungi may be an excellent system for producing various active enzymes in a single bioreactor.

## 3. Cellulolytic Enzymes and Their Regulatory Mechanisms in Fungi

### 3.1. Classification of Cellulolytic Enzymes

Cellulases are complex enzymes that degrade cellulose into cellobiose or glucose. In generally, the degradation of cellulose requires three types of enzymes: endoglucanases (EGs: EC 3.2.1.4), cellobiohydrolases (CBHs: EC 3.2.1.91), and β-glucosidases (BGLs: EC3.2.1.21) [72]. Among them, endoglucanases could cleave long cellulose into the shorter oligosaccharides; cellobiohydrolases could further degrade these shorter oligosaccharides into cellobiose; and cellobiose is finally broken down by β-glucosidases, resulting in the conversion of cellulose into D-glucose. However, some fungi also produce other enzymes to promote the decomposition of cellulose, such as cellodextrinase (EC 3.2.1.74), which can remove disaccharide (cellobiose) from the cello-oligosaccharide. In addition, cellodextrin phosphorylase (EC 2.4.1.49), cellobiose phosphorylase (EC 2.4.1.20), and cellobiose epimerase (EC 5.1.3.11) were also found to relate to the degradation of cellulose [73]. In addition to these, the lytic polysaccharide monooxygenases (LPMOs) are also considered as effective auxiliary enzymes for cellulose degradation [74,75]. These enzymes could form a cellulase system that hydrolyzes cellulose through synergistic action. In addition, the deconstruction of cell wall polymers is in a certain order. For example, an acetyl xylan esterase from *Bjerkandera adusta* is inhibited by ferulic acid, therefore the ferulate esterases gene could be expressed once the acetyl groups have been removed from hemicellulose [76]. Except for these, a set of non-hydrolytic proteins, fungal swollenins, that are homologous to canonical plant expansins, can improve the accessibility and efficiency of the enzymes involved in saccharification of cellulose substrates by loosening macrofibrils [77].

### 3.2. Mechanism of Cellulase Induction

Cellulose-degrading fungi can be directly used in industrial production, but the optimum conditions for enzyme production and utilization must be considered simultaneously, because their environmental requirements are not uniform. For example, some enzymes have maximum activity at 80 °C, while fungi often produce enzymes at the optimum temperature of 30 °C. Therefore, how to create a suitable working environment for both requires further exploration. Of course, cellulase production and its industrial applications can be separated, but this also increases the extraction and purification steps of the enzyme. Both methods have their advantages and disadvantages. At present, the main method to increase cellulase yield is through molecular biotechnology. 

The first step is for the fungi to sense an external carbon source. According to available carbon sources, the production of cellulase and xylanase is regulated at the transcriptional level in fungi, and only when plant polysaccharides (such as cellulose and xylan) are provided as carbon sources does the fungus begin to produce these enzymes in large quantities [78,79,80,81]. However, when using easily metabolized carbon sources such as glucose, the production of these enzymes is inhibited [79]. These suggested that several signal transduction pathways responsible for each of these inducers might control the expression of cellulase and xylanase. For example, the heterotrimeric G-protein GanB(alpha)-SfaD(beta)-GpgA(gamma) is a carbon source sensor that controls cAMP/PKA signaling in response to glucose [82]. The GanB may be involved in sensing various carbon sources and subsequently activating downstream signal transduction. In addition, HxtB, a glucose and xylose transporter, has been confirmed to localize to the plasma membrane and may play a role in downstream glucose signaling and metabolism [83]. Furthermore, the protein kinase PskA has an important function in the control of sugar flux and metabolism [84]. However, carbon source sensors, subsequent transport, and cellular signaling pathways still remain largely unelucidated. It has been reported that coregulation of these cellulolytic and xylanolytic enzymes can effectively degrade plant cell wall polysaccharides. Since these polymers from plants cannot enter fungal cells directly, it has been suggested that the expression of these cellulase- (or hemicellulase)-encoding genes is induced by the existence of soluble sugars degraded from cellulose [78,85,86]. The primary product of cellulose degradation by cellulase is called cellobiose. Studies have shown that cellobiose could induce the production of cellulase in many fungi, such as *T. reesei* [86,87,88]. However, cellobiose can be further hydrolyzed into glucose by extracellular β-glucosidases, and the presence of glucose inhibits the uptake of cellobiose, therefore resulting in the inhibition of cellulase expression [79,89]. Some studies have indicated that reduction in BGL activity can lead to an increasing cellulose production, such as the deletion of the extracellular BGL encoding gene or addition of the inhibitor of β-glucosidase in the media [90]. Based on these results, current molecular biology techniques have been applied to improve cellulase production.

### 3.3. Molecular Regulation Mechanisms of Cellulase Gene Expression

**Figure 1:**
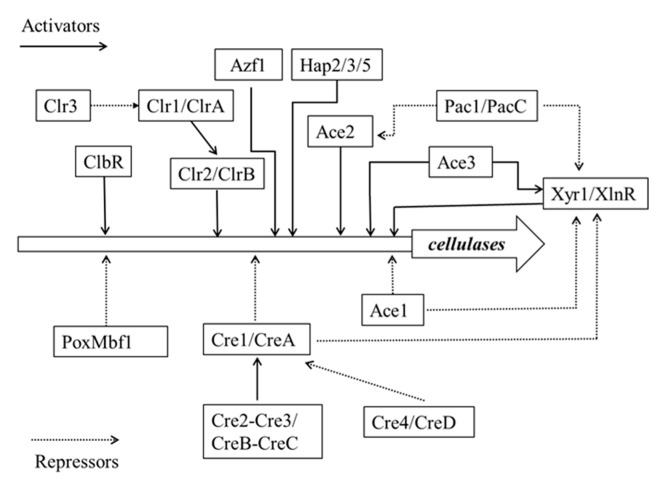
Schematic diagram of a transcriptional regulatory network of genes encoding cellulolytic enzymes in fungi. The solid line with arrowhead represents activated gene expression. The dotted line with arrowhead represents suppressed gene expression.

The efficient degradation of plant cell wall polysaccharides requires not only a wide range of cellulases to be secreted in large quantities but also a complex regulatory system to control the expression of these genes. Most of the transcription factors involved in these regulatory mechanisms belong to the Zn2Cys6 zinc binuclear cluster family, which to date has only been found to be specific to fungi [91]. Understanding of the molecular mechanism involved in cellulase and xylanase gene transcription regulation in filamentous fungi has made great progress. Through extensive analysis of these hydrolase genes and their promoter regions, several transcription regulators involved in the expression of these enzymes have been identified. The molecular mechanisms of transcription factors that regulate the expression of cellulase genes are shown in Figure 1.

XlnR, as a fungal-specific Zn2Cys6-type positive regulator, was first identified in *A. niger* [92], and then its orthologous gene was further isolated and designated Xyr1 (xylanase regulator 1) in *T. reesei* [93]. Some studies have demonstrated that it regulates the expression of not only cellulolytic genes [94,95] but also xylanolytic genes [92,95]. It has been reported that the deletion of the Xyr1 gene resulted in importantly decreased expression levels of major xylanolytic genes (*xyn1* and *xyn2*) and cellulolytic genes (*cbh1*, *cbh2*, *egl1*, and *bgl1*), even under induction culture conditions [95]. Cellulose and xylan are the most abundant polysaccharides in nature; therefore, XlnR/Xyr1 is a critical regulator in the biomass utilization process. In addition to polysaccharide degradation, XlnR/Xyr1 also regulates the expression of enzymes (D-xylose reductase, XyrA) involved in first step of the pentose catabolic pathway, which is the main pathway for the conversion of L-arabinose and D-xylose [96]. Studies in *Aspergillus* species have shown that the regulation of the pentose catabolic pathway also requires synergies with another transcription factor, AraR [97,98]. In industrial fermentation production, a large amount of hemicellulose is produced, and the presence of these hemicelluloses will inhibit the secretion of fungal cellulase. By exploring the regulation of Xyr1 on the xylanase pathway, cellulase production may be enhanced by an external hemicellulose, such as xylan as carbon source [99]. Of course, Xyr1 is not the only transcription factor that can sense the external carbon source. BglR (β-glucosidase regulator) is also involved in the sensing of cellobiose, but the specific mechanism of its action has not been thoroughly studied, and needs further exploration [100]. In addition, the transcription factor BglR has a low homology with the AmyR of *A. oryzae*, which is a key activator of amylase gene expression. 

In recent years, a novel Zn2Cys6-type fungal-specific transcription factor, ClbR, was identified to be involved in the degradation of cellulose and cellobiose in *Aspergillus aculeatus* [101]. ClbR was found to induce the expression of several cellulolytic genes, such as the FII-carboxymethyl cellulase (*cmc2*) and FIII-avicelase (*cbhI*). Additionally, it also coregulated the expression of the FIb-xylanase (*xynIb*) and FI-carboxymethyl cellulase (*cmc1*) genes with XlnR [101]. From the above studies, we can see that in addition to the genus *Trichoderma*, the molecular regulation mechanism of cellulase is also fully studied in *Aspergillus* species. 

Clr-1 is another transcription factor that is necessary for cellulose utilization. However, its regulation of cellulase gene expression is strongly influenced by inducers: Clr-1 cannot induce the expression of the cellulase gene without an inducer [102]. In the presence of an inducer, such as cellobiose, a degradation product of cellulose, Clr-1 induces the expression of some genes containing β-glucosidases and Clr-2, a transcription regulator [103]. The Clr-2 regulator was found to be responsible for the expression of major cellulolytic genes [104]. The deletion of the two genes may block cellulose utilization as a carbon source by *Neurospora crassa* [103]. Interestingly, even in the absence of inducers, Clr-2 expression leads to activation of cellulase gene expression [104]. Through the screening of mutants in the absence of an inducer, a Clr-3 that inhibits the activity of Clr-1 was identified in *N. crassa*. It was found that the deletion of the *clr*-3 gene led to cellulase gene expression of Clr-1–dependent even without an inducer [102,105].

These transcription factors often recognize and bind to specific sequences in target gene promoters. Some transcription factors are pathway specific and can be clustered in the same gene cluster with the target genes, while others have their own substrate preferences, such as activation by specific inducers or regulation by carbon catabolite repression (CCR) [106]. CCR, as a universal regulatory mechanism, is mediated by the Cre-transcription factor, which inhibits the expression of many genes by binding to specific sites in the target gene promoter region. The CreA transcriptional regulator was first identified in *Aspergillus nidulans* [107,108]. CreA inhibits the transcription of genes encoding enzymes that are involved in the polysaccharides’ degradation when in the presence of simple sugars such as glucose or fructose or other monomeric carbon sources, such as mannose or xylose. [109]. It has been reported that the CreA repressor may specifically bind to the SYGGRG sequence on the promoters of the target genes and inhibit their expression [109,110]. It is likely that this inhibitory mechanism requires further posttranslational modification of the CreA protein or interaction between proteins [111]. In *A. niger*, CreA has been shown to inhibit gene expression involved in the utilization of xylan, cellulose [109], and arabinan [112]. The final monomer product of polysaccharide degradation, such as glucose, is actually a suppressor; therefore, the entire regulatory mechanism is based on a concentration-dependent balance between transcriptional induction and CreA inhibition. In addition, *T. reesei* Cre1 was also identified and isolated as an ortholog of the *A. nidulans* CreA [113]. Studies have shown that in the presence of D-glucose, Cre1 is phosphorylated by casein kinase II-like protein, which is necessary for the DNA binding of Cre1 [114]. It has been demonstrated that the expression of *xyn1* and *cbh1* is directly regulated by the glucose repressor Cre1 [115,116]. However, Cre1 is not directly involved in the expression of *xyn2* and *cbh2* [117,118]. The CCR of cellulase and hemicellulase genes was confirmed to be mediated by Cre1 through complementary experiments in the *cre1* mutant *T. reesei* Rut C-30 by the full-length *cre1* gene [115]. In addition to Cre1, another regulator that is able to control CCR has been discovered in *T. reesei*, named Cre2. *T. reesei* Cre2 protein is an ortholog of *A. nidulans* CreB, which has been identified as an ubiquitin c-terminal hydrolase associated with the deubiquitination of Cre1 [119]. The Cre2/CreB protein has been shown to interact with the Cre3/CreC WD40-repeat protein under both carbon catabolite repressing and derepressing conditions. The interaction is necessary and may stabilize the Cre2/CreB protein by preventing its proteolysis [120]. Another member of the Cre protein family is CreD, which is involved in an opposing process to the complex of Cre2/CreB and Cre3/CreC proteins and inhibits the activity of Cre1/CreA [121].

Moreover, two other genes encoding cellulose regulators, AceI and AceII, were identified in *T. reesei* [58,122]. Of the two, AceII is a transcriptional activator of all major cellulolytic enzyme genes, including *cbh1*, *cbh2*, *egl1*, *egl2*, and xylanolytic gene *xyn2*, whereas AceI is an inhibitor of cellulase and xylanase expression [86]. To date, only the ortholog of *T. reesei* AceI has been isolated in *Aspergillus* [123]; however, the Ace2 homolog has not yet been found in the other filamentous fungi, suggesting that Ace2 is a species-specific regulator in *T. reesei* [101]. In addition, using a specific screening strategy for candidate regulators of cellulase production, the activator Ace3 was identified [59]. The overexpression of *ace3* led to the increase in cellulase gene expression, while its deletion not only resulted in markedly reduced activity of cellulase and hemicellulase but also influenced the expression of the regulator Xyr1 gene [60,124]. The growth of the strain and secretion of a large number of proteins in filamentous fungi is often affected by the ambient pH. The pH signal transduction pathway has been well investigated in these fungi, such as *A. nidulans* and *T. reesei,* and includes PacC/Pac1, a pH-responsive transcription factor [125,126], and six pal proteins. The PacC/Pac1 regulator activates alkali-expressed genes and suppresses acid-expressed genes under high pH conditions. Studies have shown that PacC/Pac1 can also promote or inhibit cellulase production in response to changes in the external environment. The deletion of the pac1 gene leads to an increase in Xyr1 activity at neutral pH. However, the effect of Pac1 on cellulase production is often masked by other regulatory mechanisms [59,127]. In addition, based on the transcriptomic profiling during solid-state and submerged fermentation, a novel transcription factor PoxMbf1 involved in cellulase production was also identified [128]. Cellulase production is determined by both the external production environment and internal genes. The CCAAT sequences are found on a wide range of fungal promoters, and the protein complex bound to CCAAT sequences was identified as hap complexes in some important filamentous fungi, such as HapB/C/E in *A. nidulans* and Hap2/3/5 in *T. reesei* [129,130]. These complexes have been confirmed to regulate the expression of some genes, including polysaccharidase genes, such as *A. oryzae* taka-amylase (*taa*) and *T. reesei* cellulase and xylanase genes (*cbh2* and *xyn2*) [118,130].

## 4. Amylolytic Enzymes and Their Regulatory Mechanisms in Fungi

### 4.1. Amylolytic Enzymes

Starch is a major storage polysaccharide in plants. It consists of multiple glucose units that are linked by α-1,4-glycosidic bonds and branched by α-1,6-glycosidic bonds. Filamentous fungi secrete a large number of starch-hydrolytic enzymes, including α-amylase, glucoamylase, and α-glucosidase, all of which are induced by starch, dextrin, or maltose. These amylolytic enzymes play a crucial role in traditional food and beverage fermentation production. Among them, α-amylase breaks down the 1,4-glycosidic linkages in starch into glucose, maltose, and other oligosaccharides. To date, three α-amylase encoding genes (*amyA/B/C*) have been first identified in *A. oryzae* [131,132,133]. Next, the glucoamylase gene *glaA* and α-glucosidase gene *agdA* were identified, respectively, in *A. oryzae* [134,135,136]. In addition, a glucoamylase gene *glaB*, which was purified from rice Koji [137], is highly induced under solid-state culture conditions and, therefore, plays a key role in solid-state fermentation. By comparing the sequences in the promoter region of these amylolytic genes, it has been found that in addition to the TATA and CCAAT sequences, there were three highly conserved regions I, II, and III potentially involved in gene expression [138,139,140]. Further deletion analysis verified that region III was crucial for the high expression of amylolytic genes. The modified promoter is constructed by introducing multiple copies of region III into the promoter regions of the *glaA* and *agdA* genes. The constructed promoters induce high-level expression of related genes downstream of the promoters, which could be used for improving the production of heterologous protein [141]. 

### 4.2. Molecular Regulation Mechanism of Amylase Gene Expression

By screening the protein binding to region III in the *amyB* promoter, a transcription factor gene *amyR* was successfully isolated [142]. AmyR is a Zn(II)2Cys6-type regulator that induces the expression of starch-degradation genes, and it has been well studied in *A. nidulans* and *A. oryzae* [142,143]. With starch as the carbon source, the *amyR* mutants displayed extremely poor growth and produced significantly fewer amylolytic enzymes, such as approximately 10-fold lower levels of glucoamylase and 100-fold lower levels of α-amylase relative to the control strain, suggesting that AmyR is importantly involved in the regulation of amylolytic enzyme gene expression in *A. oryzae* [142]. Genome sequencing analysis showed that the three genes *amyR*, *amyA*, and *agdA* constituted an amylolytic gene cluster that successively appeared in adjacent parts of the same chromosome in *A. oryzae* [41]. In addition to these two adjacent genes, AmyR has also been found to regulate the expression of α-amylase encoding gene *amyB* and glucoamylase gene *glaA* in *A. oryzae* [143]. In addition, the expression of the glucoamylase gene *glaB*, which is expressed exclusively in solid-state culture, was also regulated by the positive transcription factor AmyR, since the deletion of the *amyR* gene led to the loss of *glaB* gene expression [144]. After screening and identification of transcription factors of *glaB* genes from the mutant library of *A. oryzae*, a C_2_H_2_-type transcription factor, FlbC, was found to be involved in regulating *glaB* gene expression [145]. The disruption of the *flbC* gene caused a significant reduction in glucoamylase activity under solid-state culture conditions relative to the control. It has been reported that FlbC is also related to the regulation of conidiospore development in *Aspergillus* species [146,147]. Some research also showed that the loss of *amyR* function also indirectly affects the production of cellulolytic and hemicellulolytic enzymes in *A. oryzae* [144]. Recent studies have revealed that the basic-region helix–loop–helix (bHLH) transcription factor DevR is significantly involved in polysaccharide (such as chitin and starch) metabolism in *A. oryzae* [148]. The overexpression of *AodevR* led to a strong inhibition of strain growth and amylase-related gene expression. The yeast one-hybrid assay indicated that DevR potentially interacts with the *amyR* promoter, providing a novel insight for further revealing the regulatory mechanism of amylolytic enzyme production [148].

Interestingly, the disruption of *A. nidulans amyR* prevented growth on a medium in which starch or maltose was used as a carbon source, implying that the regulatory mechanism of gene expression under the control of AmyR is different in *A. nidulans* and *A. oryzae*. This could be explained by the existence of an additional MAL cluster involved in maltose utilization in *A. oryzae* [149]. The MAL cluster contains three genes, which encode a maltose-responsive regulator (*malR*), an intracellular α-glucosidase (*malT*), and a maltose permease (*malP*) [149,150]. The deletion of the *malR* gene led to the loss of expression of both *malT* and *malP*, suggesting that the transcription factor MalR is necessary for the expression of the two genes in *A. oryzae*. The deletion of *malR* and *malP* also caused a dramatic delay in the production of α-amylase [151]. In addition, the addition of glucose generally results in a significant decrease in amylolytic enzyme gene expression because of carbon catabolite repression (CCR), which is regulated by the negative regulator CreA [107,152], consistent with the cellulase regulatory mechanism described above.

## 5. Conclusions

Some filamentous fungi have the ability to produce and secrete large amounts of enzymes; therefore, they are widely used in the food, pharmaceutical, detergent, textile, biofuel, and other industries, especially *Trichoderma*, *Penicillium*, and *Aspergillus* strains [153,154,155]. In addition, they can use lignocellulosic waste to reduce environmental pollution. Among them, the molecular regulatory mechanism of related enzymes (such as cellulase and amylase) of the genera *Trichoderma* and *Aspergillus* has been well studied. *Aspergillus* species, which can use starch as a substrate for the traditional fermentation production of foods and beverages, have been utilized for thousands of years. However, the utilization of plant cell wall polysaccharides and the production of related enzymes (such as cellulases and hemicellulases) still remain relatively expensive for commercial application. Therefore, it is important to improve enzyme production and fermentation efficiency by screening for effective microbial species, constructing genetically engineered strains, and further selecting appropriate culture processes. Previous studies have shown that in addition to the genus *Trichoderma*, *Aspergillus* species not only secrete a large amount of amylase and protease during fermentation but also produce cellulase to make use of polysaccharides in plant cell walls. Moreover, the regulatory mechanisms for cellulase genes in *Aspergillus* species have been extensively studied. The genera *Aspergillus* and *Trichoderma* have similar enzyme gene regulation mechanisms and the ability to secrete a large number of active enzymes; therefore, coculture of multiple strains to ferment plant polysaccharides to produce useful substances is a new research direction. With the recent developments in biotechnology, these fungi will open up new prospects in the field of microbial industrial utilization.

## References

[1] Gorshkova T.A., Kozlova L.V., Mikshina P.V. (2013). Spatial structure of plant cell wall polysaccharides and its functional significance. Biochemistry.

[2] Voiniciuc C., Pauly M., Usadel B. (2018). Monitoring Polysaccharide Dynamics in the Plant Cell Wall. Plant Physiol..

[3] Lovegrove A., Edwards C.H., De Noni I., Patel H., El S.N., Grassby T., Zielke C., Ulmius M., Nilsson L., Butterworth P.J. (2017). Role of polysaccharides in food, digestion, and health. Crit. Rev. Food Sci. Nutr..

[4] Meents M.J., Watanabe Y., Samuels A.L. (2018). The cell biology of secondary cell wall biosynthesis. Ann. Bot..

[5] Chen F., Srinivasa Reddy M.S., Temple S., Jackson L., Shadle G., Dixon R.A. (2006). Multi-site genetic modulation of monolignol biosynthesis suggests new routes for formation of syringyl lignin and wall-bound ferulic acid in alfalfa (*Medicago sativa* L.). Plant J..

[6] Barros J., Serk H., Granlund I., Pesquet E. (2015). The cell biology of lignification in higher plants. Ann. Bot..

[7] Ellis M., Egelund J., Schultz C.J., Bacic A. (2010). Arabinogalactan-proteins: Key regulators at the cell surface?. Plant Physiol..

[8] Showalter A.M., Basu D. (2016). Extensin and Arabinogalactan-Protein Biosynthesis: Glycosyltransferases, Research Challenges, and Biosensors. Front. Plant Sci..

[9] Park S., Baker J.O., Himmel M.E., Parilla P.A., Johnson D.K. (2010). Cellulose crystallinity index: Measurement techniques and their impact on interpreting cellulase performance. Biotechnol. Biofuels.

[10] Moreira L.R., Filho E.X. (2016). Insights into the mechanism of enzymatic hydrolysis of xylan. Appl. Microbiol. Biotechnol..

[11] Park J., Kim B., Son J., Lee J.W. (2018). Solvo-thermal in situ transesterification of wet spent coffee grounds for the production of biodiesel. Bioresour. Technol..

[12] Yang H.P., Yan R., Chen H.P., Lee D.H., Zheng C.G. (2007). Characteristics of hemicellulose, cellulose and lignin pyrolysis. Fuel.

[13] Kumar P., Barrett D.M., Delwiche M.J., Stroeve P. (2009). Methods for Pretreatment of Lignocellulosic Biomass for Efficient Hydrolysis and Biofuel Production. Ind. Eng. Chem. Res..

[14] Taher H., Al-Zuhair S., Al-Marzouqi A.H., Haik Y., Farid M. (2014). Effective extraction of microalgae lipids from wet biomass for biodiesel production. Biomass Bioenergy.

[15] Fatma S., Hameed A., Noman M., Ahmed T., Shahid M., Tariq M., Sohail I., Tabassum R. (2018). Lignocellulosic Biomass: A Sustainable Bioenergy Source for the Future. Protein Pept. Lett..

[16] Lu Y., Li G.S., Lu Y.C., Fan X., Wei X.Y. (2017). Analytical Strategies Involved in the Detailed Componential Characterization of Biooil Produced from Lignocellulosic Biomass. Int. J. Anal. Chem..

[17] Lynd L.R., Weimer P.J., van Zyl W.H., Pretorius I.S. (2002). Microbial cellulose utilization: Fundamentals and biotechnology. Microbiol. Mol. Biol. Rev..

[18] Lee W.H., Jin Y.S. (2017). Evaluation of Ethanol Production Activity by Engineered Saccharomyces cerevisiae Fermenting Cellobiose through the Phosphorolytic Pathway in Simultaneous Saccharification and Fermentation of Cellulose. J. Microbiol. Biotechnol..

[19] Xu H.P., Li Y., Hua D.L., Mu H., Zhao Y.X., Chen G.Y. (2019). Methane production from the anaerobic digestion of substrates from corn stover: Differences between the stem bark, stem pith, and leaves. Sci. Total Environ..

[20] Gaworski M., Jablonski S., Pawlaczyk-Graja I., Ziewiecki R., Rutkowski P., Wieczynska A., Gancarz R., Lukaszewicz M. (2017). Enhancing biogas plant production using pig manure and corn silage by adding wheat straw processed with liquid hot water and steam explosion. Biotechnol. Biofuels.

[21] Chandra R., Takeuchi H., Hasegawa T. (2012). Methane production from lignocellulosic agricultural crop wastes: A review in context to second generation of biofuel production. Renew. Sust. Energy Rev..

[22] Yang B., Wyman C.E. (2008). Pretreatment: The key to unlocking low-cost cellulosic ethanol. Biofuel. Bioprod. Bior..

[23] Amin F.R., Khalid H., Zhang H., Rahman S.U., Zhang R., Liu G., Chen C. (2017). Pretreatment methods of lignocellulosic biomass for anaerobic digestion. AMB Express.

[24] Taherzadeh M.J., Karimi K. (2008). Pretreatment of lignocellulosic wastes to improve ethanol and biogas production: A review. Int. J. Mol. Sci..

[25] Ward A.J., Hobbs P.J., Holliman P.J., Jones D.L. (2008). Optimisation of the anaerobic digestion of agricultural resources. Bioresour. Technol..

[26] Shen Y., Jarboe L., Brown R., Wen Z. (2015). A thermochemical-biochemical hybrid processing of lignocellulosic biomass for producing fuels and chemicals. Biotechnol. Adv..

[27] Xia Y., Wang Y., Fang H.H., Jin T., Zhong H., Zhang T. (2014). Thermophilic microbial cellulose decomposition and methanogenesis pathways recharacterized by metatranscriptomic and metagenomic analysis. Sci. Rep..

[28] Sarparanta M., Pourat J., Carnazza K.E., Tang J., Paknejad N., Reiner T., Kostiainen M.A., Lewis J.S. (2019). Multimodality labeling strategies for the investigation of nanocrystalline cellulose biodistribution in a mouse model of breast cancer. Nucl. Med. Biol..

[29] Sharma A., Thakur M., Bhattacharya M., Mandal T., Goswami S. (2019). Commercial application of cellulose nano-composites-A review. Biotechnol. Rep..

[30] Zhang Z.Y., Sun Y., Zheng Y.D., He W., Yang Y.Y., Xie Y.J., Feng Z.X., Qiao K. (2020). A biocompatible bacterial cellulose/tannic acid composite with antibacterial and anti-biofilm activities for biomedical applications. Mater. Sci. Eng. C Mater. Biol. Appl..

[31] Xun Z., Ni S., Gao Z., Zhang Y., Gu J., Huo P. (2019). Construction of Polymer Electrolyte Based on Soybean Protein Isolate and Hydroxyethyl Cellulose for a Flexible Solid-State Supercapacitor. Polymers.

[32] Lou H.M., He X.X., Cai C., Lan T.Q., Pang Y.X., Zhou H.F., Qiu X.Q. (2019). Enhancement and Mechanism of a Lignin Amphoteric Surfactant on the Production of Cellulosic Ethanol from a High-Solid Corncob Residue. J. Agric. Food Chem..

[33] Nghiem N.P., Senske G.E., Kim T.H. (2016). Pretreatment of Corn Stover by Low Moisture Anhydrous Ammonia (LMAA) in a Pilot-Scale Reactor and Bioconversion to Fuel Ethanol and Industrial Chemicals. Appl. Biochem. Biotechnol..

[34] Rich J.O., Bischoff K.M., Leathers T.D., Anderson A.M., Liu S., Skory C.D. (2018). Resolving bacterial contamination of fuel ethanol fermentations with beneficial bacteria-An alternative to antibiotic treatment. Bioresour. Technol..

[35] Nishimura I., Shinohara Y., Oguma T., Koyama Y. (2018). Survival strategy of the salt-tolerant lactic acid bacterium, Tetragenococcus halophilus, to counteract koji mold, Aspergillus oryzae, in soy sauce brewing. Biosci. Biotechnol. Biochem..

[36] Albergaria H., Arneborg N. (2016). Dominance of Saccharomyces cerevisiae in alcoholic fermentation processes: Role of physiological fitness and microbial interactions. Appl. Microbiol. Biotechnol..

[37] Kim H.Y., Park S.S., Lim S.T. (2015). Preparation, characterization and utilization of starch nanoparticles. Colloids Surf. B Biointerfaces.

[38] Kitamoto K. (2015). Cell biology of the Koji mold Aspergillus oryzae. Biosci. Biotechnol. Biochem..

[39] Papagianni M. (2007). Advances in citric acid fermentation by Aspergillus niger: Biochemical aspects, membrane transport and modeling. Biotechnol. Adv..

[40] Kitamoto K. (2002). Molecular biology of the Koji molds. Adv. Appl. Microbiol..

[41] Machida M., Asai K., Sano M., Tanaka T., Kumagai T., Terai G., Kusumoto K.I., Arima T., Akita O., Kashiwagi Y. (2005). Genome sequencing and analysis of Aspergillus oryzae. Nature.

[42] Machida M., Yamada O., Gomi K. (2008). Genomics of Aspergillus oryzae: Learning from the History of Koji Mold and Exploration of Its Future. DNA Res..

[43] Abarca M.L., Accensi F., Cano J., Cabanes F.J. (2004). Taxonomy and significance of black aspergilli. Antonie Leeuwenhoek Int. J. G.

[44] Jin F.J., Watanabe T., Juvvadi P.R., Maruyama J.I., Arioka M., Kitamoto K. (2007). Double disruption of the proteinase genes, tppA and pepE, increases the production level of human lysozyme by Aspergillus oryzae. Appl. Microbiol. Biotechnol..

[45] Joosten V., Gouka R.J., van den Hondel C.A.M.J.J., Verrips C.T., Lokman B.C. (2005). Expression and production of llama variable heavy-chain antibody fragments (V(HH)s) by Aspergillus awamori. Appl. Microbiol. Biotechnol..

[46] Jin F.J., Katayama T., Maruyama J., Kitamoto K. (2016). Comparative genomic analysis identified a mutation related to enhanced heterologous protein production in the filamentous fungus Aspergillus oryzae. Appl. Microbiol. Biotechnol..

[47] Kim S.K., Jo J.H., Jin Y.S., Seo J.H. (2017). Enhanced ethanol fermentation by engineered Saccharomyces cerevisiae strains with high spermidine contents. Bioprocess Biosyst. Eng..

[48] Myburgh M.W., Cripwell R.A., Favaro L., van Zyl W.H. (2019). Application of industrial amylolytic yeast strains for the production of bioethanol from broken rice. Bioresour. Technol..

[49] Ruchala J., Kurylenko O.O., Dmytruk K.V., Sibirny A.A. (2019). Construction of advanced producers of first- and second-generation ethanol in Saccharomyces cerevisiae and selected species of non-conventional yeasts (Scheffersomyces stipitis, Ogataea polymorpha). J. Ind. Microbiol. Biotechnol..

[50] Beltran G., Torija M.J., Novo M., Ferrer N., Poblet M., Guillamon J.M., Rozes N., Mas A. (2002). Analysis of yeast populations during alcoholic fermentation: A six year follow-up study. Syst. Appl. Microbiol..

[51] Torija M.J., Rozes N., Poblet M., Guillamon J.M., Mas A. (2001). Yeast population dynamics in spontaneous fermentations: Comparison between two different wine-producing areas over a period of three years. Antonie Leeuwenhoek Int. J. G.

[52] Xufre A., Albergaria H., Inacio J., Spencer-Martins I., Girio F. (2006). Application of fluorescence in situ hybridisation (FISH) to the analysis of yeast population dynamics in winery and laboratory grape must fermentations. Int. J. Food Microbiol..

[53] Salmon J.M. (1989). Effect of Sugar Transport Inactivation in Saccharomyces cerevisiae on Sluggish and Stuck Enological Fermentations. Appl. Environ. Microbiol..

[54] Talamantes D., Biabini N., Dang H., Abdoun K., Berlemont R. (2016). Natural diversity of cellulases, xylanases, and chitinases in bacteria. Biotechnol Biofuels.

[55] Mandels M., Reese E.T. (1957). Induction of cellulase in Trichoderma viride as influenced by carbon sources and metals. J. Bacteriol..

[56] Ilmen M., Saloheimo A., Onnela M.L., Penttila M.E. (1997). Regulation of cellulase gene expression in the filamentous fungus Trichoderma reesei. Appl. Environ. Microbiol..

[57] Nogawa M., Goto M., Okada H., Morikawa Y. (2001). L-Sorbose induces cellulase gene transcription in the cellulolytic fungus Trichoderma reesei. Curr. Genet..

[58] Aro N., Saloheimo A., Ilmen M., Penttila M. (2001). ACEII, a novel transcriptional activator involved in regulation of cellulase and xylanase genes of Trichoderma reesei. J. Biol. Chem..

[59] Hakkinen M., Valkonen M.J., Westerholm-Parvinen A., Aro N., Arvas M., Vitikainen M., Penttila M., Saloheimo M., Pakula T.M. (2014). Screening of candidate regulators for cellulase and hemicellulase production in Trichoderma reesei and identification of a factor essential for cellulase production. Biotechnol. Biofuels.

[60] Shida Y., Furukawa T., Ogasawara W. (2016). Deciphering the molecular mechanisms behind cellulase production in Trichoderma reesei, the hyper-cellulolytic filamentous fungus. Biosci. Biotechnol. Biochem..

[61] Mantyla A.L., Rossi K.H., Vanhanen S.A., Penttila M.E., Suominen P.L., Nevalainen K.M. (1992). Electrophoretic karyotyping of wild-type and mutant Trichoderma longibrachiatum (reesei) strains. Curr. Genet..

[62] Porciuncula Jde O., Furukawa T., Mori K., Shida Y., Hirakawa H., Tashiro K., Kuhara S., Nakagawa S., Morikawa Y., Ogasawara W. (2013). Single nucleotide polymorphism analysis of a Trichoderma reesei hyper-cellulolytic mutant developed in Japan. Biosci. Biotechnol. Biochem..

[63] Shida Y., Yamaguchi K., Nitta M., Nakamura A., Takahashi M., Kidokoro S., Mori K., Tashiro K., Kuhara S., Matsuzawa T. (2015). The impact of a single-nucleotide mutation of bgl2 on cellulase induction in a Trichoderma reesei mutant. Biotechnol. Biofuels.

[64] Cohen R., Suzuki M.R., Hammel K.E. (2005). Processive endoglucanase active in crystalline cellulose hydrolysis by the brown rot basidiomycete Gloeophyllum trabeum. Appl. Environ. Microbiol..

[65] Martinez D., Larrondo L.F., Putnam N., Gelpke M.D., Huang K., Chapman J., Helfenbein K.G., Ramaiya P., Detter J.C., Larimer F. (2004). Genome sequence of the lignocellulose degrading fungus Phanerochaete chrysosporium strain RP78. Nat. Biotechnol..

[66] Steffen K.T., Cajthaml T., Snajdr J., Baldrian P. (2007). Differential degradation of oak (Quercus petraea) leaf litter by litter-decomposing basidiomycetes. Res. Microbiol..

[67] Baldrian P., Valaskova V. (2008). Degradation of cellulose by basidiomycetous fungi. FEMS Microbiol. Rev..

[68] Stursova M., Zifcakova L., Leigh M.B., Burgess R., Baldrian P. (2012). Cellulose utilization in forest litter and soil: Identification of bacterial and fungal decomposers. FEMS Microbiol. Ecol..

[69] Ransom-Jones E., Jones D.L., McCarthy A.J., McDonald J.E. (2012). The Fibrobacteres: An important phylum of cellulose-degrading bacteria. Microb. Ecol..

[70] Jiang Y.P., Duarte A.V., van den Brink J., Wiebenga A., Zou G., Wang C.S., de Vries R.P., Zhou Z.H., Benoit I. (2016). Enhancing saccharification of wheat straw by mixing enzymes from genetically-modified Trichoderma reesei and Aspergillus niger. Biotechnol. Lett..

[71] Kolasa M., Ahring B.K., Lubeck P.S., Lubeck M. (2014). Co-cultivation of Trichoderma reesei RutC30 with three black Aspergillus strains facilitates efficient hydrolysis of pretreated wheat straw and shows promises for on-site enzyme production. Bioresour. Technol..

[72] van den Brink J., de Vries R.P. (2011). Fungal enzyme sets for plant polysaccharide degradation. Appl. Microbiol. Biotechnol..

[73] Sharma A., Tewari R., Rana S.S., Soni R., Soni S.K. (2016). Cellulases: Classification, Methods of Determination and Industrial Applications. Appl. Biochem. Biotechnol..

[74] Harris P.V., Welner D., McFarland K.C., Re E., Navarro Poulsen J.C., Brown K., Salbo R., Ding H., Vlasenko E., Merino S. (2010). Stimulation of lignocellulosic biomass hydrolysis by proteins of glycoside hydrolase family 61: Structure and function of a large, enigmatic family. Biochemistry.

[75] Morgenstern I., Powlowski J., Tsang A. (2014). Fungal cellulose degradation by oxidative enzymes: From dysfunctional GH61 family to powerful lytic polysaccharide monooxygenase family. Brief. Funct. Genom..

[76] Cuervo-Soto L.I., Valdes-Garcia G., Batista-Garcia R., del Rayo Sanchez-Carbente M., Balcazar-Lopez E., Lira-Ruan V., Pastor N., Folch-Mallol J.L. (2015). Identification of a novel carbohydrate esterase from Bjerkandera adusta: Structural and function predictions through bioinformatics analysis and molecular modeling. Proteins.

[77] Santos C.A., Ferreira-Filho J.A., O’Donovan A., Gupta V.K., Tuohy M.G., Souza A.P. (2017). Production of a recombinant swollenin from Trichoderma harzianum in Escherichia coli and its potential synergistic role in biomass degradation. Microb. Cell Fact..

[78] Carle-Urioste J.C., Escobar-Vera J., El-Gogary S., Henrique-Silva F., Torigoi E., Crivellaro O., Herrera-Estrella A., El-Dorry H. (1997). Cellulase induction in Trichoderma reesei by cellulose requires its own basal expression. J. Biol. Chem..

[79] Kubicek C.P., Messner R., Gruber F., Mandels M., Kubicek-Pranz E.M. (1993). Triggering of cellulase biosynthesis by cellulose in Trichoderma reesei. Involvement of a constitutive, sophorose-inducible, glucose-inhibited beta-diglucoside permease. J. Biol. Chem..

[80] Zhou Q.X., Xu J.T., Kou Y.B., Lv X.X., Zhang X., Zhao G.L., Zhang W.X., Chen G.J., Liu W.F. (2012). Differential Involvement of beta-Glucosidases from Hypocrea jecorina in Rapid Induction of Cellulase Genes by Cellulose and Cellobiose. Eukaryot. Cell.

[81] Vazquez-Montoya E.L., Castro-Ochoa L.D., Maldonado-Mendoza I.E., Luna-Suarez S., Castro-Martinez C. (2019). Moringa straw as cellulase production inducer and cellulolytic fungi source. Rev. Argent. Microbiol..

[82] Lafon A., Seo J.A., Han K.H., Yu J.H., d’Enfert C. (2005). The heterotrimeric G-protein GanB(alpha)-SfaD(beta)-GpgA(gamma) is a carbon source sensor involved in early cAMP-dependent germination in Aspergillus nidulans. Genetics.

[83] Dos Reis T.F., Nitsche B.M., de Lima P.B., de Assis L.J., Mellado L., Harris S.D., Meyer V., Dos Santos R.A., Riano-Pachon D.M., Ries L.N. (2017). The low affinity glucose transporter HxtB is also involved in glucose signalling and metabolism in Aspergillus nidulans. Sci. Rep..

[84] Rutter J., Probst B.L., McKnight S.L. (2002). Coordinate regulation of sugar flux and translation by PAS kinase. Cell.

[85] el-Gogary S., Leite A., Crivellaro O., Eveleigh D.E., el-Dorry H. (1989). Mechanism by which cellulose triggers cellobiohydrolase I gene expression in Trichoderma reesei. Proc. Natl. Acad. Sci. USA.

[86] Aro N., Pakula T., Penttila M. (2005). Transcriptional regulation of plant cell wall degradation by filamentous fungi. FEMS Microbiol. Rev..

[87] Lin L.C., Chen Y., Li J.G., Wang S.S., Sun W.L., Tian C.G. (2017). Disruption of non-anchored cell wall protein NCW-1 promotes cellulase production by increasing cellobiose uptake in Neurospora crassa. Biotechnol. Lett..

[88] Parisutham V., Chandran S.P., Mukhopadhyay A., Lee S.K., Keasling J.D. (2017). Intracellular cellobiose metabolism and its applications in lignocellulose-based biorefineries. Bioresour. Technol..

[89] Hsieh C.W.C., Cannella D., Jorgensen H., Felby C., Thygesen L.G. (2014). Cellulase Inhibition by High Concentrations of Monosaccharides. J. Agric. Food Chem..

[90] Fowler T., Brown R.D. (1992). The bgl1 gene encoding extracellular beta-glucosidase from Trichoderma reesei is required for rapid induction of the cellulase complex. Mol. Microbiol..

[91] Brakhage A.A. (2013). Regulation of fungal secondary metabolism. Nat. Rev. Microbiol..

[92] van Peij N.N., Visser J., de Graaff L.H. (1998). Isolation and analysis of xlnR, encoding a transcriptional activator co-ordinating xylanolytic expression in Aspergillus niger. Mol. Microbiol..

[93] Rauscher R., Wurleitner E., Wacenovsky C., Aro N., Stricker A.R., Zeilinger S., Kubicek C.P., Penttila M., Mach R.L. (2006). Transcriptional regulation of xyn1, encoding xylanase I, in Hypocrea jecorina. Eukaryot. Cell.

[94] Gielkens M.M., Dekkers E., Visser J., de Graaff L.H. (1999). Two cellobiohydrolase-encoding genes from Aspergillus niger require D-xylose and the xylanolytic transcriptional activator XlnR for their expression. Appl. Environ. Microbiol..

[95] Stricker A.R., Grosstessner-Hain K., Wurleitner E., Mach R.L. (2006). Xyr1 (xylanase regulator 1) regulates both the hydrolytic enzyme system and D-xylose metabolism in Hypocrea jecorina. Eukaryot. Cell.

[96] Hasper A.A., Trindade L.M., van der Veen D., van Ooyen A.J.J., de Graaff L.H. (2004). Functional analysis of the transcriptional activator XlnR from Aspergillus niger. Microbiology.

[97] Battaglia E., Zhou M., de Vries R.P. (2014). The transcriptional activators AraR and XlnR from Aspergillus niger regulate expression of pentose catabolic and pentose phosphate pathway genes. Res. Microbiol..

[98] Ishikawa K., Kunitake E., Kawase T., Atsumi M., Noguchi Y., Ishikawa S., Ogawa M., Koyama Y., Kimura M., Kanamaru K. (2018). Comparison of the paralogous transcription factors AraR and XlnR in Aspergillus oryzae. Curr. Genet..

[99] Xiao W.J., Li H.N., Xia W.C., Yang Y.X., Hu P., Zhou S.N., Hu Y.M., Liu X.P., Dai Y.J., Jiang Z.B. (2019). Co-expression of cellulase and xylanase genes in Sacchromyces cerevisiae toward enhanced bioethanol production from corn stover. Bioengineered.

[100] Nitta M., Furukawa T., Shida Y., Mori K., Kuhara S., Morikawa Y., Ogasawara W. (2012). A new Zn(II)(2)Cys(6)-type transcription factor BglR regulates beta-glucosidase expression in Trichoderma reesei. Fungal Genet. Biol..

[101] Kunitake E., Tani S., Sumitani J., Kawaguchi T. (2013). A novel transcriptional regulator, ClbR, controls the cellobiose- and cellulose-responsive induction of cellulase and xylanase genes regulated by two distinct signaling pathways in Aspergillus aculeatus. Appl. Microbiol. Biotechnol..

[102] Huberman L.B., Coradetti S.T., Glass N.L. (2017). Network of nutrient-sensing pathways and a conserved kinase cascade integrate osmolarity and carbon sensing in Neurospora crassa. Proc. Natl. Acad. Sci. USA.

[103] Coradetti S.T., Craig J.P., Xiong Y., Shock T., Tian C., Glass N.L. (2012). Conserved and essential transcription factors for cellulase gene expression in ascomycete fungi. Proc. Natl. Acad. Sci. USA.

[104] Coradetti S.T., Xiong Y., Glass N.L. (2013). Analysis of a conserved cellulase transcriptional regulator reveals inducer-independent production of cellulolytic enzymes in Neurospora crassa. Microbiologyopen.

[105] Craig J.P., Coradetti S.T., Starr T.L., Glass N.L. (2015). Direct Target Network of the Neurospora crassa Plant Cell Wall Deconstruction Regulators CLR-1, CLR-2, and XLR-1. Mbio.

[106] Keller N.P., Turner G., Bennett J.W. (2005). Fungal secondary metabolism-From biochemistry to genomics. Nat. Rev. Microbiol..

[107] Dowzer C.E., Kelly J.M. (1989). Cloning of the creA gene from Aspergillus nidulans: A gene involved in carbon catabolite repression. Curr. Genet..

[108] Dowzer C.E., Kelly J.M. (1991). Analysis of the creA gene, a regulator of carbon catabolite repression in Aspergillus nidulans. Mol. Cell Biol..

[109] de Vries R.P., Visser J. (2001). Aspergillus enzymes involved in degradation of plant cell wall polysaccharides. Microbiol. Mol. Biol. Rev..

[110] Takashima S., Iikura H., Nakamura A., Masaki H., Uozumi T. (1996). Analysis of Cre1 binding sites in the Trichoderma reesei cbh1 upstream region. FEMS Microbiol. Lett..

[111] Roy P., Lockington R.A., Kelly J.M. (2008). CreA-mediated repression in Aspergillus nidulans does not require transcriptional auto-regulation, regulated intracellular localisation or degradation of CreA. Fungal Genet. Biol..

[112] Flipphi M.J., Visser J., van der Veen P., de Graaff L.H. (1994). Arabinase gene expression in Aspergillus niger: Indications for coordinated regulation. Microbiology.

[113] Strauss J., Mach R.L., Zeilinger S., Hartler G., Stoffler G., Wolschek M., Kubicek C.P. (1995). Cre1, the carbon catabolite repressor protein from Trichoderma reesei. FEBS Lett..

[114] Cziferszky A., Mach R.L., Kubicek C.P. (2002). Phosphorylation positively regulates DNA binding of the carbon catabolite repressor Cre1 of Hypocrea jecorina (Trichoderma reesei). J. Biol. Chem..

[115] Ilmen M., Thrane C., Penttila M. (1996). The glucose repressor gene cre1 of Trichoderma: Isolation and expression of a full-length and a truncated mutant form. Mol. Gen. Genet..

[116] Ilmen M., Onnela M.L., Klemsdal S., Keranen S., Penttila M. (1996). Functional analysis of the cellobiohydrolase I promoter of the filamentous fungus Trichoderma reesei. Mol. Gen. Genet..

[117] Zeilinger S., Mach R.L., Kubicek C.P. (1998). Two adjacent protein binding motifs in the cbh2 (cellobiohydrolase II-encoding) promoter of the fungus Hypocrea jecorina (Trichoderma reesei) cooperate in the induction by cellulose. J. Biol. Chem..

[118] Wurleitner E., Pera L., Wacenovsky C., Cziferszky A., Zeilinger S., Kubicek C.P., Mach R.L. (2003). Transcriptional regulation of xyn2 in Hypocrea jecorina. Eukaryot. Cell.

[119] Denton J.A., Kelly J.M. (2011). Disruption of Trichoderma reesei cre2, encoding an ubiquitin C-terminal hydrolase, results in increased cellulase activity. BMC Biotechnol..

[120] Lockington R.A., Kelly J.M. (2002). The WD40-repeat protein CreC interacts with and stabilizes the deubiquitinating enzyme CreB in vivo in Aspergillus nidulans. Mol. Microbiol..

[121] Boase N.A., Kelly J.M. (2004). A role for creD, a carbon catabolite repression gene from Aspergillus nidulans, in ubiquitination. Mol. Microbiol..

[122] Aro N., Ilmen M., Saloheimo A., Penttila M. (2003). ACEI of Trichoderma reesei is a repressor of cellulase and xylanase expression. Appl. Environ. Microbiol..

[123] Chilton I.J., Delaney C.E., Barham-Morris J., Fincham D.A., Hooley P., Whitehead M.P. (2008). The Aspergillus nidulans stress response transcription factor StzA is ascomycete-specific and shows species-specific polymorphisms in the C-terminal region. Mycol. Res..

[124] Xue Y., Han J., Li Y.Y., Liu J., Gan L.H., Long M.N. (2020). Promoting cellulase and hemicellulase production from Trichoderma orientalis EU7-22 by overexpression of transcription factors Xyr1 and Ace3. Bioresour. Technol..

[125] Tilburn J., Sarkar S., Widdick D.A., Espeso E.A., Orejas M., Mungroo J., Penalva M.A., Arst H.N. (1995). The Aspergillus PacC zinc finger transcription factor mediates regulation of both acid- and alkaline-expressed genes by ambient pH. EMBO J..

[126] He R.L., Ma L.J., Li C., Jia W.D., Li D.M., Zhang D.Y., Chen S.L. (2014). Trpac1, a pH response transcription regulator, is involved in cellulase gene expression in Trichoderma reesei. Enzym. Microb. Technol..

[127] Hakkinen M., Sivasiddarthan D., Aro N., Saloheimo M., Pakula T.M. (2015). The effects of extracellular pH and of the transcriptional regulator PACI on the transcriptome of Trichoderma reesei. Microb. Cell Fact..

[128] Zhao S., Liu Q., Wang J.X., Liao X.Z., Guo H., Li C.X., Zhang F.F., Liao L.S., Luo X.M., Feng J.X. (2019). Differential transcriptomic profiling of filamentous fungus during solid-state and submerged fermentation and identification of an essential regulatory gene PoxMBF1 that directly regulated cellulase and xylanase gene expression. Biotechnol. Biofuels.

[129] Brakhage A.A., Andrianopoulos A., Kato M., Steidl S., Davis M.A., Tsukagoshi N., Hynes M.J. (1999). HAP-Like CCAAT-binding complexes in filamentous fungi: Implications for biotechnology. Fungal. Genet. Biol..

[130] Zeilinger S., Ebner A., Marosits T., Mach R., Kubicek C.P. (2001). The Hypocrea jecorina HAP 2/3/5 protein complex binds to the inverted CCAAT-box (ATTGG) within the cbh2 (cellobiohydrolase II-gene) activating element. Mol. Genet. Genom..

[131] Wirsel S., Lachmund A., Wildhardt G., Ruttkowski E. (1989). Three alpha-amylase genes of Aspergillus oryzae exhibit identical intron-exon organization. Mol. Microbiol..

[132] Tsukagoshi N., Furukawa M., Nagaba H., Kirita N., Tsuboi A., Udaka S. (1989). Isolation of a cDNA encoding Aspergillus oryzae Taka-amylase A: Evidence for multiple related genes. Gene.

[133] Nemoto T., Maruyama J., Kitamoto K. (2012). Contribution ratios of amyA, amyB, amyC genes to high-level alpha-amylase expression in Aspergillus oryzae. Biosci. Biotechnol. Biochem..

[134] Hata Y., Kitamoto K., Gomi K., Kumagai C., Tamura G., Hara S. (1991). The glucoamylase cDNA from Aspergillus oryzae: Its cloning, nucleotide sequence, and expression in Saccharomyces cerevisiae. Agric Biol. Chem..

[135] Hata Y., Tsuchiya K., Kitamoto K., Gomi K., Kumagai C., Tamura G., Hara S. (1991). Nucleotide sequence and expression of the glucoamylase-encoding gene (glaA) from Aspergillus oryzae. Gene.

[136] Minetoki T., Gomi K., Kitamoto K., Kumagai C., Tamura G. (1995). Nucleotide sequence and expression of alpha-glucosidase-encoding gene (agdA) from Aspergillus oryzae. Biosci. Biotechnol. Biochem..

[137] Hata Y., Ishida H., Ichikawa E., Kawato A., Suginami K., Imayasu S. (1998). Nucleotide sequence of an alternative glucoamylase-encoding gene (glaB) expressed in solid-state culture of Aspergillus oryzae. Gene.

[138] Tada S., Gomi K., Kitamoto K., Kumagai C., Tamura G., Hara S. (1991). Identification of the promoter region of the Taka-amylase A gene required for starch induction. Agric. Biol. Chem..

[139] Tsuchiya K., Tada S., Gomi K., Kitamoto K., Kumagai C., Tamura G. (1992). Deletion analysis of the Taka-amylase A gene promoter using a homologous transformation system in Aspergillus oryzae. Biosci. Biotechnol. Biochem..

[140] Kanemori Y., Gomi K., Kitamoto K., Kumagai C., Tamura G. (1999). Insertion analysis of putative functional elements in the promoter region of the Aspergillus oryzae Taka-amylase A gene (amyB) using a heterologous Aspergillus nidulans amdS-lacZ fusion gene system. Biosci. Biotechnol. Biochem..

[141] Minetoki T., Kumagai C., Gomi K., Kitamoto K., Takahashi K. (1998). Improvement of promoter activity by the introduction of multiple copies of the conserved region III sequence, involved in the efficient expression of Aspergillus oryzae amylase-encoding genes. Appl. Microbiol. Biotechnol..

[142] Gomi K., Akeno T., Minetoki T., Ozeki K., Kumagai C., Okazaki N., Iimura Y. (2000). Molecular cloning and characterization of a transcriptional activator gene, amyR, involved in the amylolytic gene expression in Aspergillus oryzae. Biosci. Biotechnol. Biochem..

[143] Petersen K.L., Lehmbeck J., Christensen T. (1999). A new transcriptional activator for amylase genes in Aspergillus. Mol. Gen. Genet..

[144] Watanabe J., Tanaka H., Mogi Y., Yamazaki T., Suzuki K., Watanabe T., Yamada O., Akita O. (2011). Loss of Aspergillus oryzae amyR function indirectly affects hemicellulolytic and cellulolytic enzyme production. J. Biosci. Bioeng..

[145] Tanaka M., Yoshimura M., Ogawa M., Koyama Y., Shintani T., Gomi K. (2016). The C2H2-type transcription factor, FlbC, is involved in the transcriptional regulation of Aspergillus oryzae glucoamylase and protease genes specifically expressed in solid-state culture. Appl. Microbiol. Biotechnol..

[146] Kwon N.J., Garzia A., Espeso E.A., Ugalde U., Yu J.H. (2010). FlbC is a putative nuclear C2H2 transcription factor regulating development in Aspergillus nidulans. Mol. Microbiol..

[147] Ogawa M., Tokuoka M., Jin F.J., Takahashi T., Koyama Y. (2010). Genetic analysis of conidiation regulatory pathways in koji-mold Aspergillus oryzae. Fungal Genet. Biol..

[148] Zhuang M., Zhang Z.M., Jin L., Wang B.T., Koyama Y., Jin F.L. (2019). The Basic-Region Helix-Loop-Helix Transcription Factor DevR Significantly Affects Polysaccharide Metabolism in Aspergillus oryzae. Appl. Environ. Microbiol..

[149] Hasegawa S., Takizawa M., Suyama H., Shintani T., Gomi K. (2010). Characterization and expression analysis of a maltose-utilizing (MAL) cluster in Aspergillus oryzae. Fungal Genet. Biol..

[150] Needleman R.B., Kaback D.B., Dubin R.A., Perkins E.L., Rosenberg N.G., Sutherland K.A., Forrest D.B., Michels C.A. (1984). MAL6 of Saccharomyces: A complex genetic locus containing three genes required for maltose fermentation. Proc. Natl. Acad. Sci. USA.

[151] Hiramoto T., Tanaka M., Ichikawa T., Matsuura Y., Hasegawa-Shiro S., Shintani T., Gomi K. (2015). Endocytosis of a maltose permease is induced when amylolytic enzyme production is repressed in Aspergillus oryzae. Fungal Genet. Biol..

[152] Ichinose S., Tanaka M., Shintani T., Gomi K. (2014). Improved alpha-amylase production by Aspergillus oryzae after a double deletion of genes involved in carbon catabolite repression. Appl. Microbiol. Biotechnol..

[153] Florencio C., Cunha F.M., Badino A.C., Farinas C.S., Ximenes E., Ladisch M.R. (2016). Secretome analysis of Trichoderma reesei and Aspergillus niger cultivated by submerged and sequential fermentation processes: Enzyme production for sugarcane bagasse hydrolysis. Enzym. Microb. Technol..

[154] Gong W., Zhang H., Liu S., Zhang L., Gao P., Chen G., Wang L. (2015). Comparative Secretome Analysis of Aspergillus niger, Trichoderma reesei, and Penicillium oxalicum During Solid-State Fermentation. Appl. Biochem. Biotechnol..

[155] Frisvad J.C., Moller L.L.H., Larsen T.O., Kumar R., Arnau J. (2018). Safety of the fungal workhorses of industrial biotechnology: Update on the mycotoxin and secondary metabolite potential of Aspergillus niger, Aspergillus oryzae, and Trichoderma reesei. Appl. Microbiol. Biotechnol..

